# A perspective on structural and mechanistic aspects of protein *O*-fucosylation

**DOI:** 10.1107/S2053230X18004788

**Published:** 2018-07-26

**Authors:** Erandi Lira-Navarrete, Ramon Hurtado-Guerrero

**Affiliations:** a Copenhagen Center for Glycomics, Department of Cellular and Molecular Medicine, University of Copenhagen, Copenhagen, Denmark; b BIFI, University of Zaragoza, BIFI–IQFR (CSIC) Joint Unit, Mariano Esquillor s/n, Campus Rio Ebro, Edificio I+D, Zaragoza, Spain; c Fundación ARAID, Avenida de Ranillas, 50018 Zaragoza, Spain

**Keywords:** *O*-fucosylation, protein *O*-fucosyltransferases, epidermal growth factor-like repeats, EGF repeats, thrombospondin type I repeats, TSRs, enzyme mechanisms, GDP-fucose

## Abstract

Protein *O*-fucosylation is an important post-translational modification (PTM) found in cysteine-rich repeats in proteins. Protein *O*-fucosyltransferases 1 and 2 (PoFUT1 and PoFUT2) are the enzymes responsible for this PTM and selectively glycosylate specific residues in epidermal growth factor-like (EGF) repeats and thrombospondin type I repeats (TSRs), respectively. Within the past six years, crystal structures of both enzymes have been reported, revealing important information on how they recognize protein substrates and achieve catalysis. Here, the structural information available today is summarized and how PoFUT1 and PoFUT2 employ different catalytic mechanisms is discussed.

## Abstract 2

Protein *O*-fucosyltransferases 1 and 2 perform the same post-transcriptional modification albeit on different substrates. Correct folding of the substrates is essential for protein recognition, and the crystal structures of these proteins have shown the mechanism of how these two proteins are highly specific.

## 1. Introduction   

Fucose is an important biological sugar that can be found as part of various glycoconjugates and is one of the two monosaccharides present in mammals with an l-configuration (Bertozzi & Rabuka, 2009 ▸). Fucose modifications of proteins, mostly known as *O*-fucosylation, play multiple roles in cellular events. In mammals, there are 13 glycosyltransferases (GTs) that are capable of adding a fucose residue using GDP-fucose as the sugar donor, but only two GTs, protein *O*-fucosyltransferases 1 and 2 (PoFUT1 and PoFUT2), can directly glycosylate protein side chains (Schneider *et al.*, 2017 ▸). Both PoFUT1 and PoFUT2 transfer fucose from GDP-fucose to serine or threonine residues in cysteine-rich repeats in proteins. However, while PoFUT1 glycosylates epidermal growth factor-like (EGF) repeats within the consensus sequence C^2^-*X*-*X*-*X*-*X*-S/T-C^3^ (Haltom & Jafar-Nejad, 2015 ▸), PoFUT2 glycosylates thrombospondin type I repeats (TSRs) containing Ser/Thr residues located in the consensus sequences C^1^-*X*-*X*-S/T-C^2^ or C^2^-*X*-*X*-S/T-C^3^ of TSRs of groups 1 and 2 (see below), respectively (Schneider *et al.*, 2017 ▸).

EGF repeats are small domains ranging between 30 and 40 amino acids, characterized by the formation of three disulfide bridges with the arrangement C^1^–C^3^, C^2^–C^4^ and C^5^–C^6^ (Savage *et al.*, 1973 ▸). TSRs are larger than EGF repeats (∼60 amino acids) and can be split into two groups owing to their disulfide-bridge arrangement. The disulfide bridges of group 1 TSR are arranged as C^1^–C^5^, C^2^–C^6^ and C^3^–C^4^, while TSRs of group 2 adopt the pattern C^1^–C^4^, C^2^–C^5^ and C^3^–C^6^ (Leonhard-Melief & Haltiwanger, 2010 ▸). Although the disulfide-bridge arrangement differs between the two groups, the C^2^–C^6^ and C^3^–C^6^ disulfide bridges are conserved (Leonhard-Melief & Haltiwanger, 2010 ▸). PoFUT1 and PoFUT2 both require correctly folded repeats for *O*-fucosylation to take place (Luo, Nita-Lazar *et al.*, 2006 ▸; Wang & Spellman, 1998 ▸) and both isoforms are highly selective for each repeat (Luo, Koles *et al.*, 2006 ▸; Luo, Nita-Lazar *et al.*, 2006 ▸), suggesting that the different disulfide-bridge arrangements and consensus sequences are essential features for substrate recognition. This review outlines recent progress in unveiling the differences in the reaction mechanisms and protein-substrate recognition of these enzymes, and the importance of *O*-fucosylation in protein–protein interaction.

## 2. PoFUT1 and PoFUT2 protein substrates   

The first substrate of PoFUT1 to be identified was the urinary type 1 plasminogen activator (Kentzer *et al.*, 1990 ▸). Now, approximately 100 proteins with EGF repeats are predicted to be *O*-fucosylated, although only a few have been confirmed (for a thorough review, see Schneider *et al.*, 2017 ▸). The Notch receptors, which are transmembrane type I proteins that form part of the Notch signalling pathway, are the most studied PoFUT1 substrates. Most of the EGF repeats present in the four Notch receptors found in mammals enclose the consensus sequence required for *O*-fucosylation by PoFUT1 (Takeuchi & Haltiwanger, 2014 ▸). Notch ligands, two in *Drosophila* (Delta and Serrate) and three Delta-like and two Serrate-like ligands in mammals (Dll1, Dll3, Dll4, Jagged1 and Jagged2; D’Souza *et al.*, 2008 ▸), also contain EGF repeats that can be *O*-fucosylated by PoFUT1 (Schneider *et al.*, 2017 ▸).

**Figure 1:**
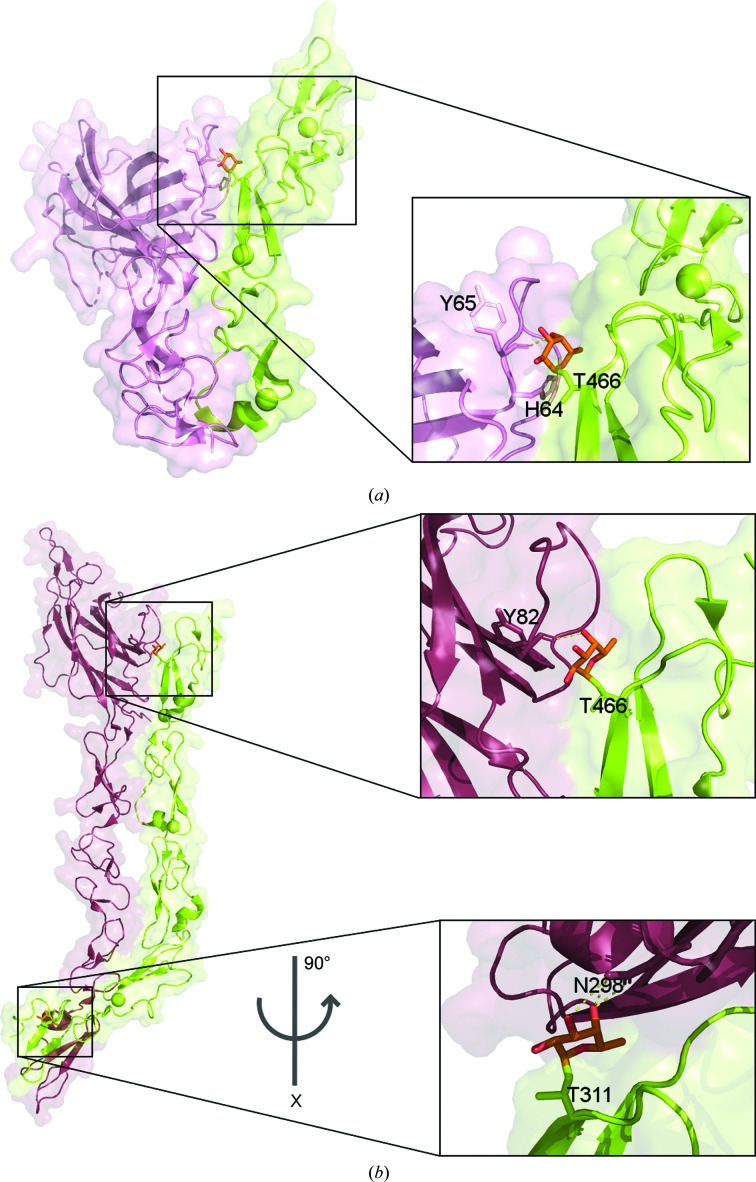
Importance of Notch fucosylation for ligand interaction. (*a*) Surface and cartoon representations of the Notch1–DLL4 complex (PDB entry 4xl1; Luca *et al.*, 2015 ▸). Notch1 EGFs 11–13 are shown in green and the MNNL-DSL-EGF1 Delta domains are shown in pink. The fucose (orange) modification of Notch1 Thr466 and its interactions with DLL4 residues are depicted in the left panel. (*b*) Surface and cartoon representation of the Notch1–Jagged1 complex (PDB entry 5uk5; Luca *et al.*, 2017 ▸). Notch 1 EGFs 8–12 are shown in green, the C2-DSL-EGF1–3 Jagged domains are shown in red and Notch fucose is shown in orange. The interactions of the Notch1 fucose modifications of Thr466 and Thr311 are depicted in the upper and lower panels, respectively. Ca^2+^ ions are shown as green spheres.

Notch glycosylation is an elegant example of how the cell uses protein glycosylation to tune signalling-pathway activity. The elongation of Notch1 *O*-fucose by the addition of an *N*-acetylglucosamine (GlcNAc) moiety by Fringe GTs directs the specificity of the Notch receptors towards Delta and reduces Notch activation by Jagged proteins (Xu *et al.*, 2007 ▸). Recent crystallo­graphic complexes between Notch1 and its ligands (DLL4 and Jagged) show that the fucose moiety also plays an essential role in ligand interaction (Luca *et al.*, 2015 ▸, 2017 ▸). The *O*-fucose moiety at Notch1 EGF12 Thr466 contributes significantly to recognizing DLL4 by hydrogen-bonding and hydrophobic interactions with the DLL4 MNNL domain (module at the N-terminus of the Notch ligand; Fig. 1 ▸ *a*; Luca *et al.*, 2015 ▸). However, the complex between Notch1 and Jagged1 is stabilized by interactions between Notch1 EGF12 Thr466-*O*-fucose and the Jagged1 C2 domain and also between Notch1 EGF8 Thr311-*O*-fucose and Jagged1 EGF3 (Fig. 1 ▸ *b*). These interactions between Jagged1 and Notch1 provide an explanation of why Jagged1 binds sixfold more tightly to Notch1 EGFs 8–12 than a construct containing only EGFs 11–12 (Luca *et al.*, 2017 ▸). Notch signalling plays a significant role in cell development, and its malfunction can lead to several diseases, including various types of cancer (Allenspach *et al.*, 2002 ▸). The glycosylation of Notch receptors may also play a role in aberrant signalling (Takeuchi & Haltiwanger, 2014 ▸), and in this sense it has been reported that PoFUT1 is overexpressed in some cancer types (Kroes *et al.*, 2007 ▸; Loo *et al.*, 2013 ▸; Yokota *et al.*, 2013 ▸).

Despite the prediction of more than 50 protein substrates, very little is known about the TSR *O*-fucose function (Schneider *et al.*, 2017 ▸). The first *O*-fucosylated TSRs described in the literature were found on thrombospondin-1 (TSP1; Hofsteenge *et al.*, 2001 ▸). Subsequently, PoFUT2 was isolated and characterized, following the realization that PoFUT1 does not glycosylate TSRs (Luo, Nita-Lazar *et al.*, 2006 ▸; Luo, Koles *et al.*, 2006 ▸). ADAMT and ADAMT-like proteins are a large family of proteins that are predicted to have several TSRs that are potentially *O*-fucosylated by PoFUT2 (Kelwick *et al.*, 2015 ▸; Schneider *et al.*, 2017 ▸), and *O*-fucose is a requirement for the efficient secretion of some of these proteins (Ricketts *et al.*, 2007 ▸; Wang *et al.*, 2007 ▸; Vasudevan *et al.*, 2015 ▸; Benz *et al.*, 2016 ▸). Very recently, it has been reported that depletion of PoFUT2 in *Plasmodium falciparum* results in attenuated infection of the mosquito vector and human hepatocytes. The authors concluded that this effect was owing to the loss of trafficking of PoFUT2 target proteins (Lopaticki *et al.*, 2017 ▸).

## 3. The PoFUT1/2 GDP-fucose binding site is highly conserved   

The first reported crystal structures of protein *O*-fucosyltransferases were those of *Caenorhabditis elegans* PoFUT1 (*Ce*PoFUT1) in the unliganded form (PDB entry 3zy4) and in complex with GDP and GDP-fucose (PDB entries 3zy3 and 3zy6) (Lira-Navarrete *et al.*, 2011 ▸). The human PoFUT1 (*Hs*PoFUT1) crystal structure was reported in the free form and in the presence of GDP-fucose (PDB entries 5ux6 and 5uxh; Li *et al.*, 2017 ▸), and crystal structures of *Mus musculus* PoFUT1 (*Mm*PoFUT1) in complex with different EGF repeats (PDB entries 5kxh, 5ky0, 5ky2, 5ky3, 5ky4, 5ky5, 5ky7, 5ky8 and 5ky9) have also been reported (Li *et al.*, 2017 ▸). Regarding PoFUT2, there are two crystal structures available for human PoFUT2: in the free form and complexed with GDP-fucose (PDB entries 4ap5 and 4ap6; Chen *et al.*, 2012 ▸). In addition, the ternary complex between *Ce*PoFUT2, GDP and the human TSR1 (*Hs*TSR1) repeat (PDB entry 5foe) has been described. Note that the latter complex is the only structure of PoFUT2 known to include an acceptor-protein substrate (Valero-González *et al.*, 2016 ▸).

**Figure 2:**
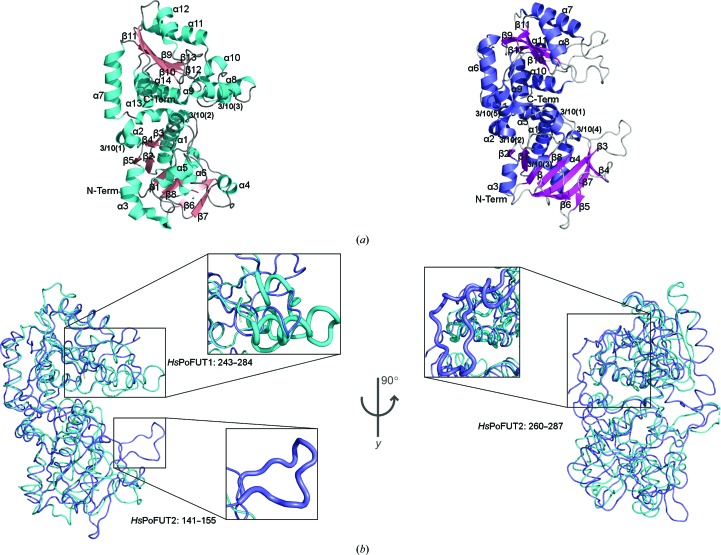
(*a*) Cartoon representations of the human PoFUT1 (PDB entry 5ux6; McMillan *et al.*, 2017 ▸) and PoFUT2 (PDB entry 4ap5; Chen *et al.*, 2012 ▸) structures. Secondary structures are shown for *Hs*PoFUT1 with helices in cyan and β-sheets in salmon; for *Hs*PoFUT2 helices are shown in slate blue and β-sheets in magenta. (*b*) Superimposed structures of *Hs*PoFUT1 (cyan) and *Hs*PoFUT2 (slate blue). Main secondary-structure differences are highlighted in boxes.

Both PoFUT1 and PoFUT2 present the typical GT-B fold, which consists of two Rossmann-like domains facing each other with the active site lying within the resulting deep cleft formed between them (Fig. 2 ▸ *a*; Lairson *et al.*, 2008 ▸). Although both enzymes share the same type of folding, the superimposition of apo forms of human PoFUT1 and PoFUT2 renders a poor root-mean-square deviation (r.m.s.d.) on 174 equivalent C^α^ atoms of 3.03 Å (Fig. 2 ▸ *b*), which is in agreement with the observed low sequence identity (∼28% identity). This difference between the *Hs*PoFUT1 and *Hs*PoFUT2 crystal structures is mainly attributed to the presence of two prominent loops in *Hs*PoFUT2 that are absent in *Hs*PoFUT1. While loop_260–287_ is located in the C-terminal domain with no apparent function in catalysis, loop_141–155_ contributes to the formation of the cleft in which the acceptor substrate is located (Fig. 2 ▸ *b*). *Hs*PoFUT1 also has additional secondary elements that are formed by residues Ser243–Leu284, which encompass three α-helices and a 3_10_-helix (Fig. 2 ▸ *b*). This region prevents the exposure of GDP-fucose to the solvent and avoids the binding of a TSR. Similarly, *Hs*PoFUT2 employs loop_141–155_ to selectively bind TSRs in contraposition to EGF repeat acceptor substrates (Valero-González *et al.*, 2016 ▸; Li *et al.*, 2017 ▸). Superimposition of both human enzymes complexed with GDP-fucose renders an r.m.s.d. on 167 equivalent C^α^ atoms of 2.62 Å, suggesting a higher fold similarity of the enzymes in the presence of the sugar donor in contrast to the free form.

**Figure 3:**
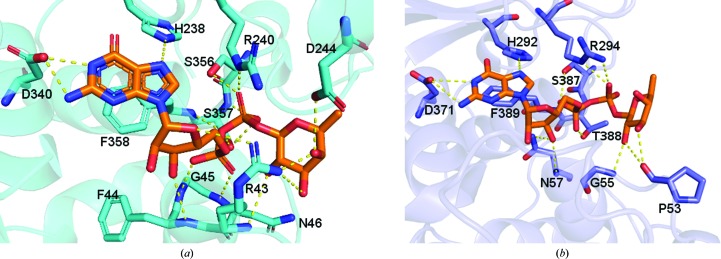
Comparison of the GDP-fucose binding site in (*a*) *Hs*PoFUT1 (PDB entry 5uxh; McMillan *et al.*, 2017 ▸) and (*b*) *Hs*PoFUT2 (PDB entry 4ap6; Chen *et al.*, 2012 ▸). GDP-fucose is shown in stick representation with orange C atoms. Amino acids of *Hs*PoFUT1 and *Hs*PoFUT2 that interact with GDP-fucose are represented as sticks with cyan and slate blue C atoms, respectively.

For both enzymes, most of the residues involved in interaction with GDP-fucose are conserved. The essential residues Arg240*^Hs^* ^PoFUT1^/Arg294*^Hs^* ^PoFUT2^ interact with the β-phosphate of GDP-fucose through hydrogen-bonding and electrostatic interactions, which are conserved in other fucosyltransferases (Martinez-Duncker *et al.*, 2003 ▸; Okajima *et al.*, 2005 ▸; Lira-Navarrete *et al.*, 2011 ▸; Chen *et al.*, 2012 ▸; McMillan *et al.*, 2017 ▸). The GDP moiety is tethered by additional inter­actions with Asn46/Asn57, His238/His292, Asp340/Asp371, Ser356/Ser387, Ser357/Thr388 and Phe358/Phe389 of *Hs*PoFUT1 and *Hs*PoFUT2, respectively (Fig. 3 ▸). GDP also interacts with the backbones of Phe44 and Gly45 in *Hs*PoFUT1. Contrary to the high level of conservation between the interacting residues of *Hs*PoFUT1 and *Hs*PoFUT2 with the GDP moiety, the residues recognizing the fucose moiety are not conserved. In particular, the fucose moiety is stabilized by interactions with Arg43/Asp244 of *Hs*PoFUT1 and Pro53/Gly55 of *Hs*PoFUT2 (Fig. 3 ▸).

## 4. PoFUT1 and PoFUT2 deploy different strategies for protein-substrate recognition   

**Figure 4:**
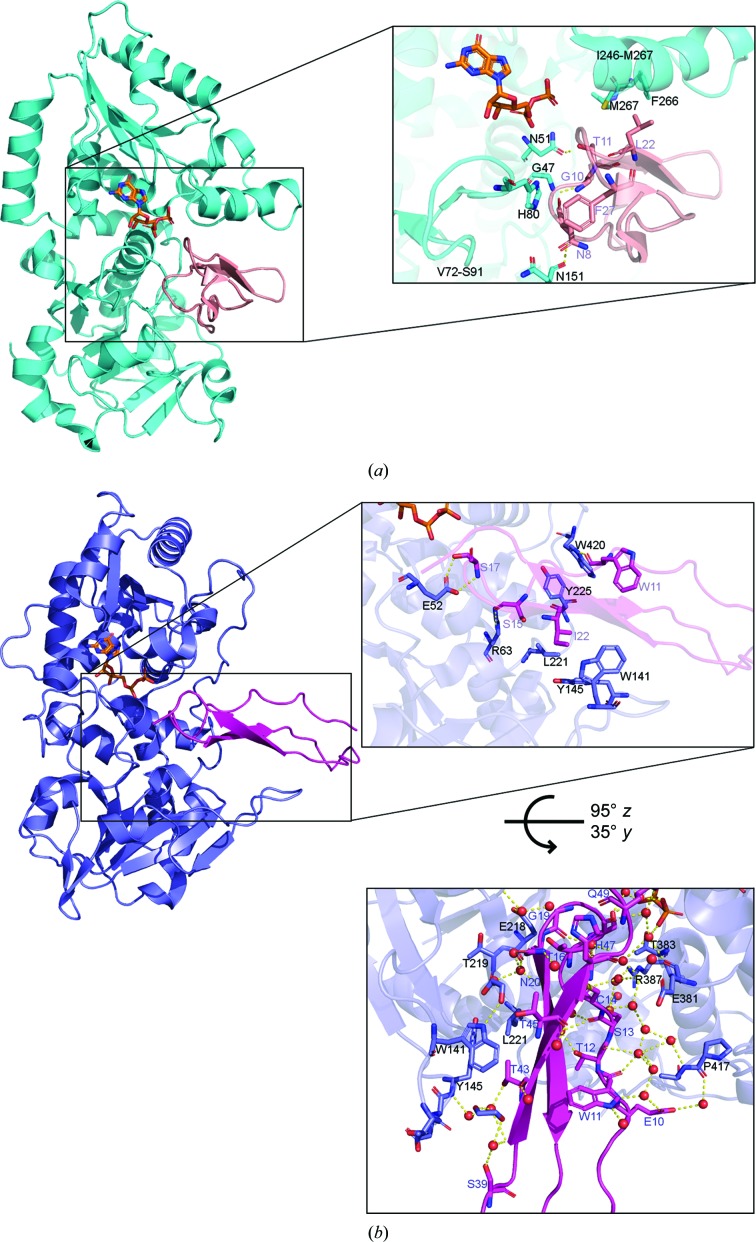
Comparison of the PoFUT1 and PoFUT2 acceptor-binding sites. (*a*) Left: cartoon representation of *Mm*PoFUT1 (cyan) in complex with *Mm*EGF26 (salmon) and GDP (depicted as sticks with orange C atoms; PDB entry 5ky4; Li *et al.*, 2017 ▸). Right: close-up view of the EFG binding site. The interacting amino acids of *Mm*PoFUT1 and *Mm*EGF26 are shown as sticks with cyan and salmon C atoms, respectively. (*b*) Left: cartoon representation of *Ce*PoFUT2 (slate blue) in complex with *Hs*TSR1 (magenta) and GDP (sticks with orange C atoms; PDB entry 5foe; Valero-González *et al.*, 2016 ▸). Upper right box: close-up view of the TSR binding site. C atoms of interacting residues are shown as sticks in slate blue (*Ce*PoFUT2) and magenta (*Hs*TSR1). Lower right box: close-up view of the TSR binding site rotated 95° around the *z* axis and 35° around the *y* axis. Amino-acid colours are the same as in the upper panel and water molecules are shown as red spheres.

The structures of both enzymes in complex with acceptor substrates highlight significant differences. Complexes between *Mm*PoFUT1 and four different EGF repeats reveal that the binding mode of all repeats is preserved (Li *et al.*, 2017 ▸). The EGF repeats locate near a hairpin formed by amino acids Val72–Ser91, which are highly conserved among different species (Lira-Navarrete *et al.*, 2011 ▸). This particular hairpin moves to maintain contact with the EGF C^5^–C^6^ subdomain through hydrophobic interactions with residues His80 or Phe85 (Li *et al.*, 2017 ▸). Other conserved interactions are made by *Mm*PoFUT1 residues Phe266 and Met267 and an apolar residue located next to the fourth cysteine in the EGF repeats (Fig. 4 ▸ *a*; Li *et al.*, 2017 ▸). Finally, primary interactions between *Mm*PoFUT1 and the different EGF repeats are formed by amino acids from the inner part of the *Mm*PoFUT1 groove and the EGF consensus sequence. Within the consensus sequence, the hydrogen-bond interaction between the acceptor Ser/Thr and Asn51*^Mm^* ^PoFUT1^ is of the utmost importance for catalytic purposes (Li *et al.*, 2017 ▸).

Looking at the *Ce*PoFUT2–GDP–*Hs*TSR1 ternary complex, it is evident that both the interactions between the *Ce*PoFUT2–*Hs*TSR1 and *Mm*PoFUT1–EGF complexes and the arrangement of the TSR and EGF repeats differ (Valero-González *et al.*, 2016 ▸; Li *et al.*, 2017 ▸). The *Ce*PoFUT2–*Hs*TSR1 complex is partly supported by direct interactions between two hydrophobic patches of *Ce*PoFUT2 and nonconserved residues of *Hs*TSR1 (Fig. 4 ▸ *b*). Three of the ten direct interactions between *Ce*PoFUT2 and *Hs*TSR1 are conserved for other TSRs, suggesting that the complex is stabilized by a limited number of direct interactions. Within these three interactions, the hydrogen bond between the acceptor Ser or Thr and the catalytic base Glu52 is essential for catalysis (Valero-González *et al.*, 2016 ▸).

A striking difference between the *Mm*PoFUT1–EGF and *Ce*PoFUT2–*Hs*TSR1 complexes is the large number of water molecules that are present in the interface of the latter. These water molecules mediate an important number of interactions through hydrogen bonds between the enzyme and *Hs*TSR1 (Fig. 4 ▸ *b*; Valero-González *et al.*, 2016 ▸). The interactions provide an explanation at the molecular level of how PoFUT2 recognizes multiple dissimilar TSRs (Leonhard-Melief & Haltiwanger, 2010 ▸; Kakuda & Haltiwanger, 2014 ▸). Therefore, both PoFUT1 and PoFUT2 deploy different strategies to identify their acceptor-protein substrates. PoFUT2 recognizes TSRs by using a limited number of direct conserved inter­actions complemented by a large number of hydrogen-bond interactions mediated by water molecules (Valero-González *et al.*, 2016 ▸). Meanwhile, PoFUT1 uses a water-filled cavity to accommodate the EGF loop C^1^–C^2^ (Li *et al.*, 2017 ▸), although the main interactions rely on conserved direct hydrogen bonds between the enzyme and its protein substrate.

## 5. Catalytic mechanisms of PoFUT1 and PoFUT2   

**Figure 5:**
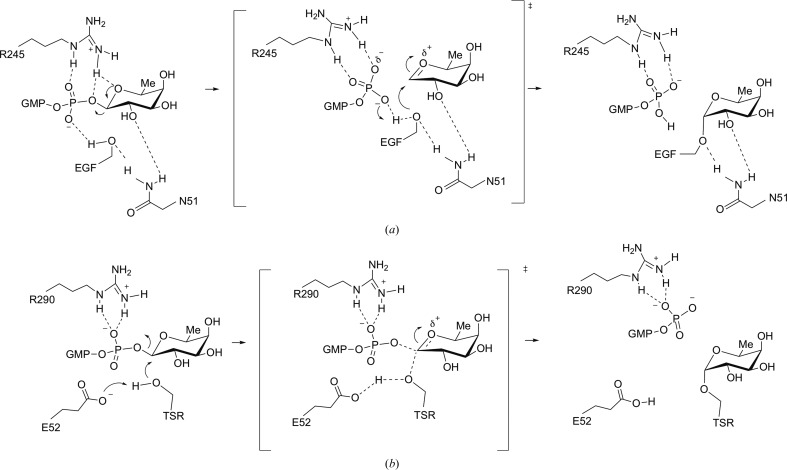
Catalytic mechanisms of PoFUT1 and PoFUT2. (*a*) The S_N_1-like catalytic mechanism proposed for PoFUT1. Note that a water-mediated proton transfer might also promote the above mechanism. (*b*) The S_N_2-like mechanism proposed for PoFUT2.

The ternary complexes also provided a better understanding of how the two enzymes achieve catalysis. These proteins are inverting GTs, implying that the acceptor Ser/Thr makes a nucleophilic attack on the nucleotide sugar anomeric C atom from the opposite side to the leaving nucleotide. As a result, this action inverts the anomeric stereochemistry (Lairson *et al.*, 2008 ▸). In this mechanism, deprotonation of the acceptor hydroxyl group by a catalytic base, usually an aspartate or a glutamate, is required to increase the nucleophilic character of the acceptor residue and is a prior step to the attack of the acceptor on the anomeric C atom (Lairson *et al.*, 2008 ▸; Breton *et al.*, 2012 ▸). While an amino acid acting as a catalytic base is present in PoFUT2 (Glu54/Glu52 in human PoFUT2 and *Ce*PoFUT2, respectively), an equivalent residue is not found in PoFUT1. As expected, mutating Glu54 and Arg294 in *Hs*PoFUT2 revealed that these two amino acids are essential for catalytic activity (Chen *et al.*, 2012 ▸). Furthermore, the *Ce*PoFUT2–GDP–*Hs*TSR1 complex, together with molecular dynamics, supports the role of a glutamate as the catalytic base. In this crystal structure, Glu52 was engaged in a hydrogen bond to the acceptor serine in the *Hs*TSR1 repeat (Valero-González *et al.*, 2016 ▸). Overall, these data support an S_N_2-like mechanism for PoFUT2 (Fig. 5 ▸ *b*), which is the typical mechanism reported for most inverting glycosyltransferases (Lairson *et al.*, 2008 ▸).

In contrast, an S_N_1-like mechanism was proposed for PoFUT1 (Fig. 5 ▸ *a*) in which Asn43 of *Ce*PoFUT1 positions the incoming acceptor and Arg240 facilitates the cleavage of the glycosidic bond by interacting with the β-phosphate group (Lira-Navarrete *et al.*, 2011 ▸). Michaelis complexes of *Mm*PoFUT1 also support an S_N_1-like mechanism and confirm a similar arrangement as above. The equivalent residues in *Mm*PoFUT1, Asn51 and Arg245, are engaged in hydrogen bonds to the acceptor residue and the β-phosphate group, respectively. Arg240*^Ce^* ^PoFUT1^/Arg245*^Mm^* ^PoFUT1^ could also favour the reaction by stabilizing GDP. In addition, the acceptor hydroxyl group is close to a water molecule that is engaged in a hydrogen bond to the β-phosphate O atom. In concordance with an S_N_1-like mechanism, this water molecule could promote catalysis by providing a proton relay serving to shuttle the acceptor hydroxyl proton to the GDP β-phosphate O atom, which acts as the catalytic base (Li *et al.*, 2017 ▸). This reaction mechanism of PoFUT1 differs from that proposed for PoFUT2, and consists of prior cleavage of the glycosidic bond, followed by attack of the pre-activated acceptor hydroxyl group on the anomeric C atom.

## 6. Final remarks   

PoFUT1 and PoFUT2 are glycosyltransferases that share great resemblances, including catalysis of the same type of PTM, architecture and the recognition of proteins with cysteine-rich repeats (Chen *et al.*, 2012 ▸; Schneider *et al.*, 2017 ▸). However, significant differences are present at the primary-structure and secondary-structure levels, accounting for the different arrangements of EGF repeats and TSRs in the binding site, the recognition mode of EGF repeats and TSRs, and the reaction mechanism. These differences explain why these enzymes serve specific substrates and are not capable of cross-recognizing their acceptor substrates. PoFUT2 has evolved to glycosylate two different groups of TSRs (Kakuda & Haltiwanger, 2014 ▸). In doing so, PoFUT2 employs a different strategy to recognize its TSRs by using a water-molecule network that mediates enzyme–protein substrate interactions (Valero-González *et al.*, 2016 ▸). In addition, while the S_N_2-like mechanism of PoFUT2 is well accepted for inverting GTs, the atypical S_N_1-like mechanism proposed for PoFUT1 needs further validation by additional experiments.

We expect that these findings will be able to be leveraged for the development of inhibitors/modulators of PoFUT1/2 that would be useful for providing further insights into the role of this PTM in animal models and for diseases and pathologies associated with these enzymes.
